# Asymptomatic versus symptomatic SARS-CoV-2 infection: a cross-sectional seroprevalence study

**DOI:** 10.1186/s41182-022-00490-9

**Published:** 2022-12-27

**Authors:** Engy Mohamed El-Ghitany, Mona H. Hashish, Azza Galal Farghaly, Eman A. Omran, Nermin A. Osman, Marwa M. Fekry

**Affiliations:** 1 Department of Tropical Health, High Institute of Public Health, Alexandria University, Alexandria, Egypt; 2 Department of Microbiology, High Institute of Public Health, Alexandria University, Alexandria, Egypt; 3 Biomedical Informatics and Medical Statistics Department, Medical Research Institute, Alexandria University, Alexandria, Egypt

**Keywords:** SARS-CoV-2, Anti-S, Anti-N, Asymptomatic SARS-CoV-2 infection

## Abstract

**Background:**

Although symptomatic SARS-CoV-2 infection predisposes patients to develop complications, the asymptomatic SARS-CoV-2 infection state is of public health importance being a hidden source of infection. Moreover, the asymptomatic state may camouflage the actual burden of the disease.

**Methods:**

Data of 1434 seropositive participants for SARS-CoV-2 spike (anti-S) and/or nucleocapsid antibodies (anti-N) were retrieved from a larger cross-sectional survey on COVID-19. Relevant data were retrieved from records including socio-demographic, medical, and behavioral characteristics of seropositive participants as well as history of COVID-19 symptoms during the last 6 months. Symptomatic/asymptomatic SARS-CoV-2 infection was categorized based on the history of the presence or absence of COVID-19 symptoms.

**Results:**

The rate of asymptomatic SARS-CoV-2 infection was 34.9%. There was a statistically significant difference between symptomatic and asymptomatic participants regarding age, residence, medical conditions, habits, and infection control measures. The number of symptoms was positively correlated with anti-S titer and both were positively correlated with adult body mass index. Slum areas residence, client-facing occupation or being a healthcare worker, having lung disease, having blood group type A, never practicing exercise or social distancing, never using soap for hand washing, and minimal engagement in online working/studying were independent factors associated with the symptomatic state. Patients having less than three symptoms were less likely to be diagnosed by any means.

**Conclusions:**

One-third of SARS-CoV-2 infections in our study were asymptomatic. This mandates applying proper measures to prevent transmission even from apparently healthy individuals. Modifiable factors associated with symptomatic infection should be controlled to reduce the risk of COVID-19 complications.

## Introduction

Coronavirus disease 2019 (COVID-19), the disease caused by severe acute respiratory syndrome coronavirus 2 (SARS-CoV-2), was declared by the World Health Organization (WHO) as a pandemic on 11 March 2020 [1]. On February 14, 2020, Egypt announced the first case of COVID-19, and three months later, the number of cases reached 10,000 [2]. The initial wave of the pandemic in Egypt was dominated by lineage B viruses (mainly B.1), while the second and third waves were dominated by C-like lineages (especially C.36) [3].

SARS-CoV-2 infection may be asymptomatic or may present with a wide spectrum of symptoms, ranging from fever, cough and dyspnea to severe pneumonia and acute respiratory distress syndrome, requiring supplemental oxygen and mechanical ventilation. Both asymptomatic and symptomatic states of SARS-CoV-2 infection carry significance in the dynamics of the COVID-19 pandemic. Individuals with asymptomatic SARS-CoV-2 infection have positive serological tests (anti-nucleocapsid and/or anti-spike) but no symptoms, thus they may act as a hidden source of infection. Therefore, diagnosing asymptomatic infections is critical for constraining SARS-CoV-2 transmission. On the other hand, among symptomatic patients, some factors are reported to be associated with a worse prognosis among COVID-19 patients. Identification of such factors would help in the prevention of complications among high-risk individuals [4].

Different plans of action have been carried out by health authorities as well as decision-makers to manage this pandemic [5]. Although the detection of asymptomatic patients may help in controlling subsequent outbreaks, scarce data are available regarding factors affecting the development of asymptomatic infection [6]. Therefore, this work aimed to identify such factors and to determine the rate of asymptomatic infection for better insights into the magnitude of COVID-19 within the Egyptian population.

## Methods

A total of 1434 participants were retrieved from the records of a previous larger cross-sectional survey on COVID-19. That survey took place during the period between January and June 2021, which coincided with the second and third waves of the COVID-19 pandemic in Egypt. At that time, COVID-19 vaccines were restricted for healthcare workers. The detailed sample size, sampling technique and data collection methods and tools [7], and laboratory tests for SARS-CoV-2 spike (anti-S) and/or nucleocapsid antibodies (anti-N) were described in two related publications extracted from the same funded project [7, 8].

Seropositive samples were selected for our study (positive for either anti-S and/or anti-N). Socio-demographic and clinical data of participants were recorded, as well as personal habits including social distancing, wearing masks, exercising, working online, and others. Body weight and height were measured, and body mass index (BMI) was calculated for children according to the World Health Organization charts [9], and for adults by dividing weight (kg) by the square of height (m^2^). The crowding index for each participant was calculated as the total number of co-residents per household, excluding the newborn infant, divided by the total number of rooms, excluding the kitchen and bathrooms [10]. The smoking index was calculated as the number of cigarettes smoked per day × years of tobacco use [11]. A direct slide hemagglutination test was performed for each participant to determine the participant’s blood group.

Subjects were classified as either symptomatic or asymptomatic depending on the history of presence or absence of symptoms related to SARS-CoV-2 infection (fever, cough, diarrhea, dyspnea, loss of taste/smell, runny nose, fatigue, myalgia/arthralgia) within 6 months before collection of samples. History of diagnosis of confirmed SARS-CoV-2 infection was recorded in symptomatic patients, based on the results of one or more of the following items, as decided by the treating physician: laboratory and/or radiological evidence (D-dimer, serum ferritin, complete blood picture, PCR testing for SARS-CoV-2, rapid antigen detection and chest computed tomography. The number of symptoms as well as the number of diagnosed SARS-CoV-2 infections was recorded for each participant. The duration between diagnosis and sampling was recorded (months), as well as the duration required for clinical improvement (days).

### Data analysis

Statistical analysis was carried out using SPSS statistics software version 24 (SPSS, Inc., Chicago, IL). Quantitative data were tested for normality using the Kolmogorov–Smirnov test. Qualitative data were expressed by numbers and percentages. The proportional differences between the different groups were assessed using Pearson’s Chi-square test. Spearman’s correlation was used to test the association between the study variables. Logistic regression was conducted after testing the assumptions of the predictors given that we included those that exerted significant results between asymptomatic and symptomatic cases. Roc curve analysis was submitted using the Med-Calc program to test the sensitivity, specificity, and cutoff value which are the fundamental tools for test evaluation. The optimal cutoff point was defined as the minimum value of (1 − sensitivity)^2^ + (1 − specificity)^2^. In all other applied statistical tests of significance, the* p-*value (< 0.05) was considered significant.

## Results

The enrolled participants with positive anti-S and/or anti-N comprised 1434 individuals and were grouped into 934 (65.1%) symptomatic and 500 (34.9%) asymptomatic participants depending on the presence or absence of COVID-19 symptoms. The majority of participants (*n* = 505, 35.2%) were in the age category “40–59” years. The majority of adults received a university education (40.4%) followed by “secondary education” (32.1%) while 17.05% were “illiterate”. Our participants were from ten different Egyptian governorates, with 37.2% of them from Alexandria Governorate (Table 1). Most of the participants were females (60.5%), married (76.6%), and from urban areas (57.8%).

**Table 1:** Distribution of the symptomatic and asymptomatic participants to socio-demographic data

	Total *n* = 1434 (100%)	Symptomatic *n* = 934 (65.13%)	Asymptomatic *n* = 500 (34.87%)	Statistical test	*p*-value
Age					
< 15 years	246 (17.2)	128 (52.0)	118 (48.0)	30.984	< 0.001**
15–29	244 (17.0)	167 (68.4)	77 (31.6)		
30–40	295(20.6)	219 (74.2)	76 (25.8)		
40–59	505 (35.2)	330 (65.3)	175 (34.7)		
> 60 years	144(10.0)	90 (62.5)	54 (37.5)		
Sex					
Male	567 (39.5)	360 (63.5)	207 (36.5)	1.111	0.292
Female	867 (60.5)	574 (66.2)	293 (33.8)		
Marital status	N = 1130	n = 773	n = 357		
Single	148 (13.1)	112 (75.7)	36 (24.3)	5.331	0.070
Married	866 (76.6)	588 (67.9)	278 (32.1)		
Divorced	116 (10.3)	73 (62.9)	43 (37.1)		
Residence					
Urban	829 (57.8)	563 (67.9)	266 (32.1)	7.586	0.023*
Rural	452 (31.5)	282 (62.4)	170 (37.6)		
Slum	153 (10.7)	89 (58.2)	64 (41.8)		
Occupation					
Not working	301 (21.0)	191(63.5)	110 (36.5)		
Client facing role	457 (31.9)	302 (66.1)	155 (33.9)	27.295	< 0.001*
Non-client facing role	59 (4.1)	43 (72.9)	16 (27.1)		
Health care worker	58 (4.0)	38 (65.5)	20 (34.5)		
Retired	253 (17.7)	191 (75.5)	62 (24.5)		
Students	306 (21.3)	169 (55.2)	137 (44.8)		
Crowding index					
< 1	988 (68.9)	660 (66.8)	328 (33.2)	5.390	0.068
1– < 2	394 (27.5)	246 (62.4)	148 (37.6)		
2 +	52 (3.6)	28 (53.8)	24 (46.2)		

A significant statistical difference between symptomatic and asymptomatic participants was seen between different age categories (*p* < 0.001). The proportion of symptomatic SARS-CoV-2 infection was significantly more in all age categories except the age group ≤ 15 years. The highest proportion of asymptomatic participants was among children less than 15 years old. There was also a significant difference between symptomatic and asymptomatic participants regarding their residence (rural versus urban (*p* = 0.023).

As shown in Table 2, there was a significant difference between symptomatic and asymptomatic participants concerning kidney, lung [allergy/chronic obstructive pulmonary disease (COPD)], and autoimmune diseases (*p* = 0.016, 0.001, and 0.017, respectively). There was a significantly higher proportion of participants suffering from at least one of these comorbidities among symptomatic cases compared to asymptomatic ones.

**Table 2:** Distribution of the symptomatic and asymptomatic participants in relation to their medical data

	Total *n* (%) 1434 (100)	Symptomatic *n* (%) 934 (65.13)	Asymptomatic *n* (%) 500 (34.87)	Statistical test	*p*-value
BMI for children based on WHO	(*N* = 304)	(*N* = 161)	(*N* = 143)		
Underweight (< 5th percentile)	15 (4.9)	10 (66.7)	5 (33.3)		
Normal (5th to < 85th percentile)	147 (48.4)	75 (51.0)	72 (49.0)	1.481	*0.687*
Overweight (85th to < 95th percentile)	45 (14.8)	25 (55.6)	20 (44.4)		
Obese (> 95th percentile)	97 (31.9)	51 (52.6)	46 (47.4)		
BMI for adults based on WHO	(*N* = 1130)	(*N* = 773)	(*N* = 357)		
Underweight (> 18.5)	5 (0.4)	4 (80.0)	1 (20.0)		
Normal (18.5–24.9)	198 (17.5)	137 (69.2)	61 (30.8)	0.706	*0.872*
Overweight (25–29.9)	368 (32.6)	247 (67.1)	121 (32.9)		
Obese (≥ 30)	559 (49.5)	385 (68.9)	174 (31.1)		
Smoking status					
No	1268 (88.4)	824 (65.0)	444 (35.0)	0.106	0.745
Yes	166 (11.6)	110 (66.3)	56 (33.7)		
Diabetes					
No	1286 (89.7)	834 (64.9)	452 (35.1)	0.431	0.512
Yes	148 (10.3)	100 (67.6)	48 (32.4)		
Hypertension					
No	1219 (85.0)	789 (64.7)	430 (35.3)	0.594	0.441
Yes	215 (15.0)	145 (67.4)	70 (32.6)		
Cardiac diseases					
No	1372 (95.7)	889 (64.8)	483 (35.2)	1.583	0.208
Yes	62 (4.3)	45 (72.6)	17 (27.4)		
Kidney diseases					
No	1403 (98.0)	909 (64.7)	496 (35.3)	5.788	0.016*
Yes	29 (2.0)	25 (86.2)	4 (13.8)		
Liver diseases					
No	1390 (97.0)	903 (65.0)	487 (35.0)	0.566	0.452
Yes	44 (3.0)	31 (70.5)	13 (29.5)		
Lung diseases					
No	1394 (97.2%)	898 (64.4%)	496 (35.6)	11.204	0.001*
Yes	40 (2.8%)	36 (90.0%)	4 (10.0)		
Autoimmune diseases					
No	1368 (95.4)	882 (64.5)	486 (35.5)	5.681	0.017*
Yes	66 (4.6)	52 (78.8)	14 (21.2)		
Blood group					
A	514 (35.8)	278 (63.8)	158 (36.2)		
B	387 (27.0)	207 (63.7)	118 (36.3)	1.695	0.638
AB	126 (8.8)	67 (65.7)	35 (34.3)		
O	407 (28.4)	213 (60.2)	141 (39.8)		
Rh					
Negative	98 (6.8)	60 (61.2)	38 (38.8)	0.707	0.400
Positive	1336 (93.2)	874 (65.4)	462 (34.6)		

Regarding the participants' habits and the infection control measures they adopt (Table 3), a significant statistical difference between both groups was seen regarding wearing masks, applying social distancing, using soap for hand washing, performing an exercise, and working online (*p* = 0.029, 0.036, 0.032 and 0.047, respectively).

**Table 3:** Distribution of the symptomatic and asymptomatic participants in relation to their habits and applied infection control measures

	Total n (%) 1434 (100)	Symptomatic n (%) 934 (65.13)	Asymptomatic n (%) 500 (34.87)	Statistical test	*p*-value
Wearing masks/social distancing					
Never	158 (11.0)	88 (55.7)	70 (44.3)	7.080	0.029*
Sometimes	930 (64.9)	614 (66.0)	316 (34.0)		
Always	346 (24.1)	232 (67.1)	114 (32.9)		
Washing hands after returning home					
Never	317 (22.1)	219 (69.1)	98 (30.9)	2.800	*0.094*
Sometimes	1117 (77.9)	715 (64.0)	402 (36.0)		
Use of soap for hand wash					
Never	26 (1.8)	21 (80.8)	5 (19.2)	6.670	0.036*
Sometimes	218 (15.2)	154 (70.6)	64 (29.4)		
Always	1190 (83.0)	759 (63.8)	431 (36.2)		
Use of hand disinfectant					
Never	1018 (71.0)	664 (65.2)	354 (34.8)	0.013	*0.908*
Sometimes	416 (29.0)	270 (64.9)	146 (35.1)		
Eating outdoors					
Never	1172 (81.7)	754 (64.2)	419 (35.8)	2.204	*0.138*
Sometimes	262 (18.3)	181 (69.1)	81 (30.9)		
Performing exercises					
Never	242 (16.9)	158 (65.3)	84 (34.7)	6.873	0.032*
Occasionally	476 (33.2)	331 (69.5)	145 (30.5)		
Consistently	716 (49.9)	445 (62.2)	271 (37.8)		
Regular use of public transportation					
No	120 (8.4)	82 (68.3)	38 (31.7)	0.591	*0.442*
Yes	1314 (91.6)	852 (64.8)	462 (35.2)		
Regular use of air conditioner					
No	1160 (80.9)	755 (80.8)	405 (34.9)	0.006	*0.940*
Yes	274 (19.1)	179 (65.3)	95 (34.7)		
Number of days per week working online	*N* = 552	*N* = 397	*N* = 185		
1–3	154 (27.9)	112 (72.7)	42 (27.3)	6.096	0.047*
4–5	136 (24.6)	94 (69.1)	42 (30.9)		
6–7	262 (47.5)	161 (61.5)	101 (38.5)		

Among the 934 symptomatic participants, 439 (47.01%) had 1–2 symptoms, while 290 (31.04) had 3–4 symptoms and 205 (21.95%) had 5 or more symptoms. Only 149 (16%) were diagnosed with COVID-19 among symptomatic participants. The majority of diagnosed participants (*n* = 144, 96.6%) were diagnosed only once. It was found that 56.4% of those diagnosed have been sampled within 3 months from diagnosis, 22.2% within 4–6 months, 15.4% within 7–9 months, and 6.0% within 10 months or more. Improvement from symptoms had occurred in 20.8%, 41.6%, and 37.6% of those diagnosed within 1–6, 7–14, and 15–30 days, respectively.

**Fig. 1:**
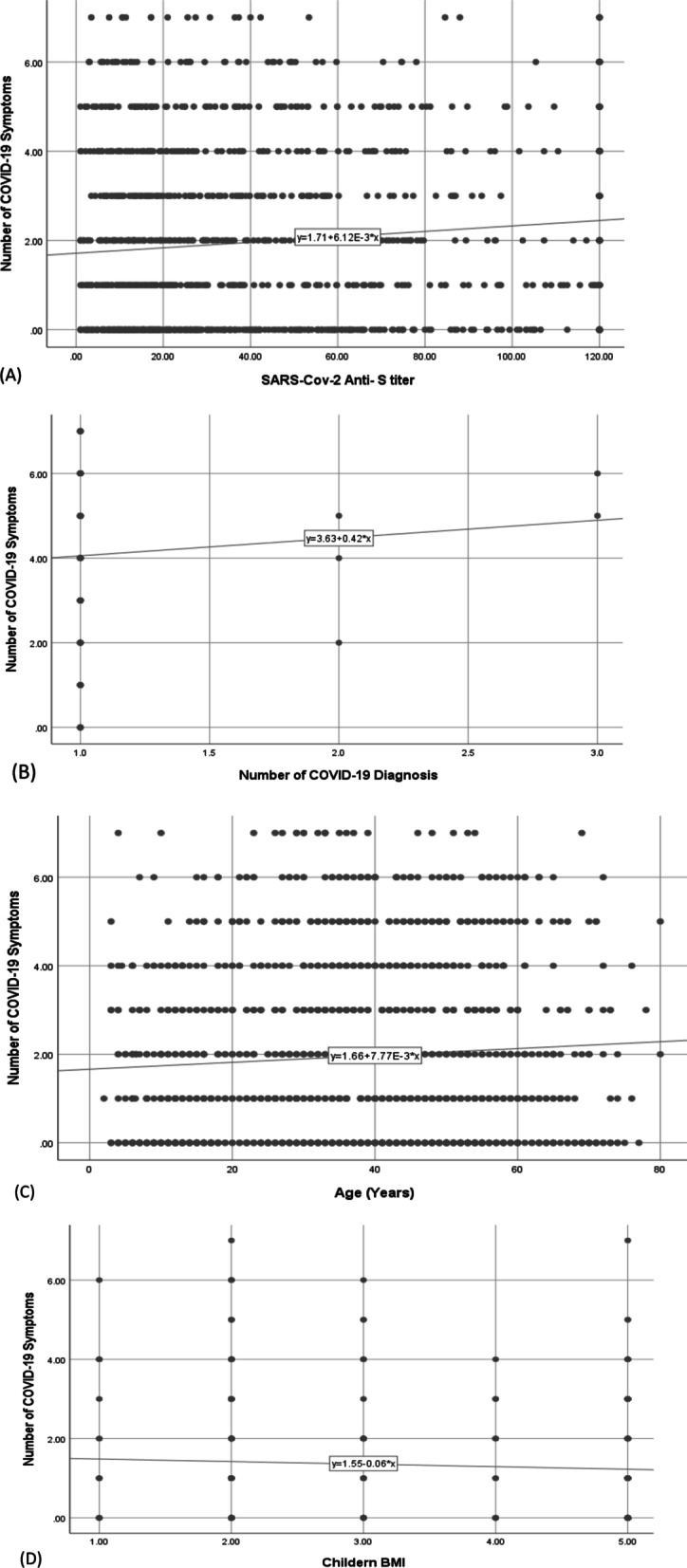

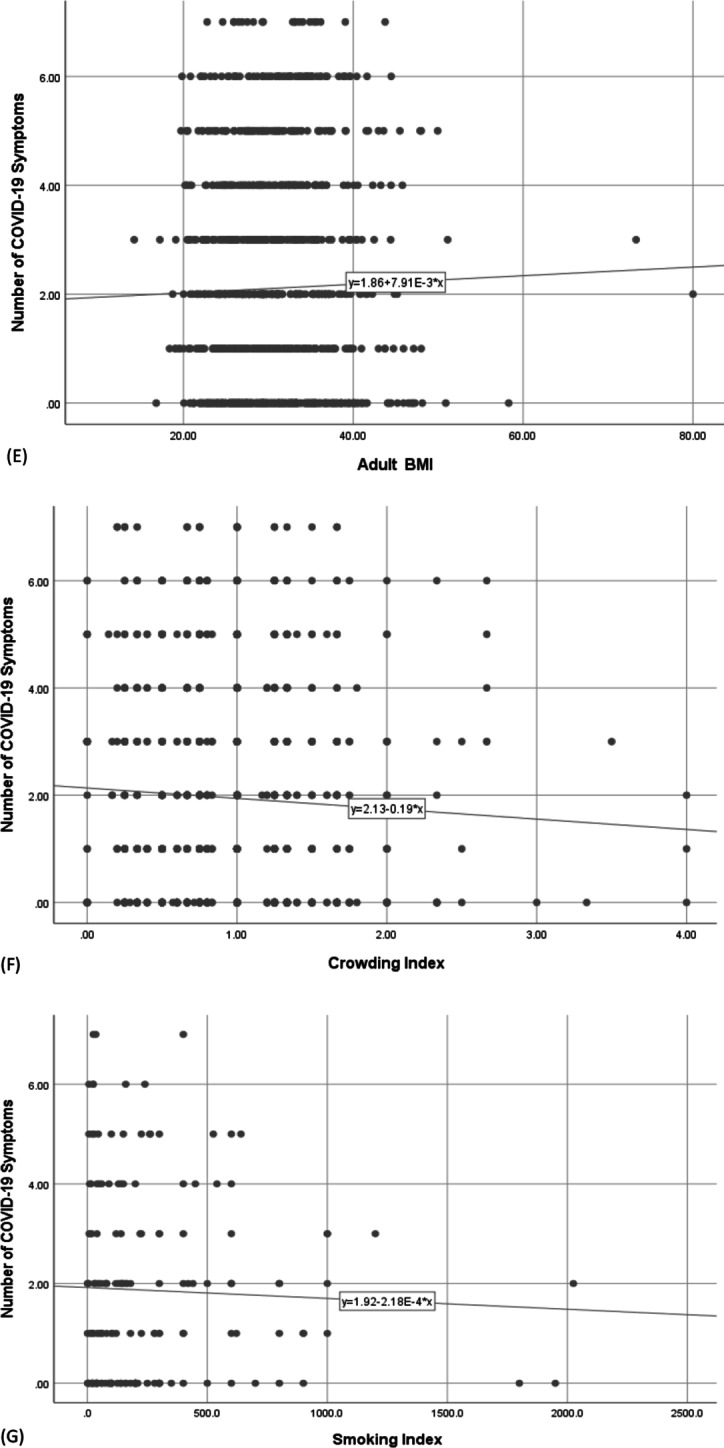
Scatter plots showing correlations between the number of symptoms and: **A** anti-S titer, **B** number of times of COVID-19 diagnosis, **C** age (years), **D** children BMI, **E** adult BMI, **F** crowding index, **G** smoking index

Using scatter plots and a correlation matrix, the number of symptoms was significantly and positively correlated with anti-S and age (*r* = 0.108 and 0.061, respectively) (Fig. 1 and Table 4). Both the number of symptoms and anti-S titer were significantly and positively correlated with adult body mass index (BMI) (*r* = 0.174). Both of them were also significantly but negatively correlated with the number of online working days (*r* = − 0.161 and − 0.126, respectively). The number of days required for improvement was significantly and positively correlated with the number of symptoms, age, and anti-S titer (*r* = 0.186, 0.240, and 0.200, respectively). Anti-S titer was significantly and negatively correlated with the duration since the diagnosis of COVID-19 (*r* = − 0.250).

**Table 4:** Correlation matrix between some quantitative factors among the studied participants

		1	2	3	4	5	6	7	8	9	10	11
Spearman’s rho												
1	R	1.000	0.108**	0.030	0.186*	− 0.317**	0.061*	− 0.004	0.174**	− 0.017	0.106	− 0.161**
	*P*	NA	< *0.001*	0.714	0.023	< *0.001*	0.022	0.949	< *0.001*	0.561	0.172	< *0.001*
2	r	0.108**	1.000	0.006	0.200*	− 0.250**	0.039	0.038	0.062	− 0.047	0.093	− 0.126**
	*p*	< *0.001*	NA	0.949	0.019	0.003	0.159	0.531	0.054	0.122	0.259	0.005
3	r	0.030	0.006	1.000	0.052	0.064	− 0.023	NA	− 0.032	− 0.016	NA	0.095
	*p*	0.714	0.949	NA	0.528	0.441	0.785	NA	0.712	0.850	NA	0.374
4	r	0.186*	0.200*	0.052	1.000	0.127	0.240**	0.730	− 0.024	− 0.047	0.000	− 0.018
	*p*	0.023	0.019	0.528	NA	0.124	0.003	0.099	0.782	0.566	1.000	0.869
5	r	− 0.317**	− 0.250**	0.064	0.127	1.000	− 0.089	− 0.483	− 0.014	0.007	0.032	0.041
	*p*	< *0.001*	0.003	0.441	0.124	NA	0.278	0.331	0.874	0.936	0.925	0.701
6	r	0.061*	0.039	− 0.023	0.240**	− 0.089	1.000	− 0.154**	− 0.295**	− 0.303**	0.418**	− 0.101*
	*p*	0.022	0.159	0.785	0.003	0.278	NA	0.007	< *0.001*	< *0.001*	< *0.001*	0.018
7	r	− 0.004	0.038	NA	0.730	− 0.483	− 0.154**	1.000	NA	− 0.100	NA	− 0.212
	*p*	0.949	0.531	NA	0.099	0.331	0.007	NA	NA	0.082	NA	0.383
8	r	0.174**	0.062	− 0.032	− 0.024	− 0.014	− 0.295**	NA	1.000	− 0.080*	− 0.025	− 0.252**
	*p*	< *0.001*	0.054	0.712	0.782	0.874	< *0.001*	NA	NA	0.019	0.757	< *0.001*
9	r	− 0.017	− 0.047	− 0.016	− 0.047	0.007	− 0.303**	− 0.100	− 0.080*	1.000	0.100	0.052
	*p*	0.561	0.122	0.850	0.566	0.936	< *0.001*	0.082	0.019	NA	0.211	0.222
10	r	0.106	0.093	NA	0.000	0.032	0.418**	NA	− 0.025	0.100	1.000	− 0.141
	*p*	0.172	0.259	NA	1.000	0.925	< *0.001*	NA	0.757	0.211	NA	0.113
11	r	− 0.161**	− 0.126**	0.095	− 0.018	0.041	− 0.101*	− 0.212	− 0.252**	0.052	− 0.141	1.000
	*p*	< *0.001*	0.005	0.374	0.869	0.701	0.018	0.383	< *0.001*	0.222	0.113	. NA

NA: not analyzed

1-Number of symptoms

2-Anti-S titer

3-Frequency of COVID-19 diagnosed infections

4-Duration after which patient improved (days)

5-Duration since SARS-CoV-2 infection (months)

6-Age (years)

7-WHO CHILD BMI

8- WHO ADULT BMI

9-Crowding index

10-Smoking index

11-Number of online working days

**Fig. 2:**
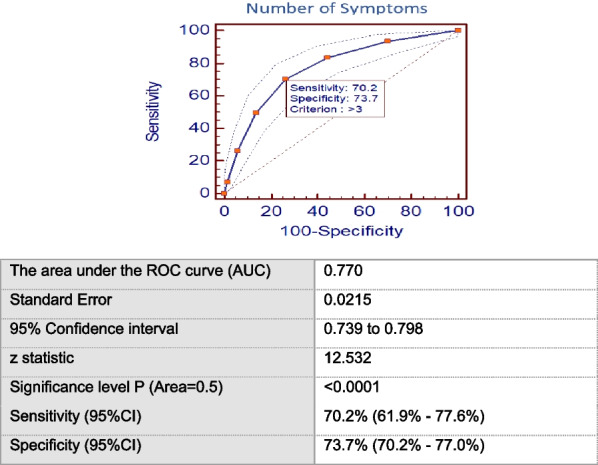
ROC curve to determine the cutoff number of symptoms at which the cases became diagnosed (*n* = 149) among the symptomatic cases (*n* = 934)

ROC analysis was used to determine the cutoff number of symptoms at which the cases became diagnosed among the symptomatic cases. It was found that having more than three symptoms was a good indicator for COVID-19 diagnosis among the symptomatic cases with a sensitivity of 70.2% [95% CI 61.9–77.6%], and specificity of 73.7% [95% CI 70.2–77.0%] as shown in Fig. 2.

Table 5 shows a multivariate logistic regression analysis for the parameters affecting the symptomatic state in the studied COVID-19 participants. All the variables that had a significance level of ≤ 0.05 in the bivariate analysis were included in the multivariate logistic regression model. Overall, the model was significant [*χ*^2^ = 43.714,* p* = 0.004*].

**Table 5:** Multivariate logistic regression analysis for the parameters affecting the symptomatic state in COVID-19 participants

	B	S.E	Sig	Adjusted odds ratio (aOR)	95% C.I. for EXP(B)	
					Lower	Upper
Residence						
Residence (1)—rural	0.626	0.338	0.064	1.870	0.964	3.627
Residence (2)—slum	1.612	0.358	0.037*	1.845	1.715	3.720
Type of occupation						
Type of occupation (1)—client-facing room	1.208	0.381	0.026*	1.231	1.083	2.597
Type of occupation (2)—health care workers	1.747	0.486	0.014*	2.111	1.914	5.475
Type of occupation (3)—retired	0.702	0.705	0.082	2.052	1.844	2.459
Kidney disease (1)	0.970	0.816	0.134	0.379	0.077	1.875
Lung disease (allergy/COPD) (1)	1.308	0.809	0.006*	2.270	2.055	3.321
Autoimmune disease (1)	0.692	0.494	0.161	0.500	0.190	1.317
Blood group						
Blood group (1)—AB	0.293	0.236	0.214	1.341	0.844	2.130
Blood group (2)—A	0.915	0.252	0.015*	1.851	1.130	3.031
Blood group (3)—B	0.550	0.367	0.134	1.734	0.844	3.563
Rh (1)	0.317	0.359	0.378	1.373	0.679	2.774
Practicing social distancing						
Practice social distancing (1): sometimes	− 0.245	0.382	0.522	0.783	0.370	1.656
Practice social distancing (2): never	1.154	0.225	0.019*	1.566	1.750	1.813
Work days/ study days per week						
Work days/study days per week (1): 1–3	0.474	0.239	0.047*	1.607	1.006	2.566
Work days/ study days per week (2): 4–5	0.265	0.240	0.268	1.304	0.815	2.085
Using soap for hand washing (1): sometimes	0.302	0.305	0.322	1.352	0.744	2.459
Using soap for hand washing (2): never	1.015	0.152	0.018*	2.851	2.130	3.931
Practicing exercise						
Practicing exercise (1)—occasionally	0.144	0.331	0.663	1.155	0.604	2.208
Practicing exercise (2)—never	0.468	0.227	0.039*	1.597	1.023	2.492
Constant	1.658	1.346	0.218	5.249		

Dependent variable: being symptomatic

Predictor(s) entered in the model: Residence (Reference: Urban), Type of occupation (Reference: Not working/Non-client Facing role), Do you suffer from kidney disease? (Reference: No), Do you have lung disease (allergy/ COPD)? (Reference: No), Do you have an autoimmune disease? (Reference: No), Blood group (Reference: Blood Group (O)), RH (Reference: Negative), Do you practice social distancing? (Reference: Always), Work days/study days [online] per week (Reference: 6–7), Do you use soap for hand washing? (Reference: Always), Do you exercise? (Reference: Consistently)

Overall model is significant [*X*^*2*^ = *43.714, p* = 0.004*]

Residence, type of occupation, kidney, lung, and autoimmune diseases, blood group, Rh, social distancing, work days/study days [online] per week, use of soap for hand washing, and exercises were included in the initial model. The most significant risk factors for symptomatic state among enrolled participants were “never using soap for hand wash” (aOR = 2.851, *p* = 0.018) followed by “presence of lung disease” (aOR = 2.27, *p* = 0.006), and being a “HCW” (aOR = 2.11, *p* = 0.014). All these parameters had approximately a 2- to 3-fold higher risk of being symptomatic. “Living in slum areas” and “having blood group A” had approximately twofold higher risk (aOR = 1.845, *p* = 0.037, and aOR = 1.851, *p* = 0.015, respectively). Other parameters such as "never performing exercises", “never performing social distancing” and “online studying or working for 1–3 days only/week” were approximately 1.5 times riskier for being symptomatic rather than asymptomatic.

## Discussion

Globally, the percentages of asymptomatic infections by SARS-CoV-2 were reported to range from 6 to 41% [12]. The prevalence of asymptomatic SARS-CoV-2 infections is of considerable concern for public health policymakers in managing the pandemic. In this work, the prevalence of asymptomatic participants was high but still within the reported range (34.9%). An outranged prevalence of asymptomatic participants (52% and 43%) was recorded from India and the United Arab Emirates, respectively [13, 14]. However, a study in England indicated that only a substantial portion (19%) of those who had detectable antibodies to SARS-CoV-2 were entirely asymptomatic [15]. Similar results were demonstrated among a sample of the Mexican population where asymptomatic patients constituted only 14.7% of the positive close contacts [16]. Two different studies from Saudi Arabia showed that asymptomatic patients represented lower percentages (7.9 and 9.3%) of the studied population [17, 18]. This wide range in prevalence of asymptomatic infections may be attributed to demographic differences in study populations, study definitions of true asymptomatic state, and variability in sensitivity and specificity of diagnostic tools.

In this study, there was a significant positive correlation between levels of anti-S titer and the appearance of symptoms among the studied participants (Table 4). The anti-S titer was significantly higher in symptomatic compared to asymptomatic participants. Similarly, other researchers reported that SARS-CoV-2-specific IgG antibody titers gradually increased with the increasing severity of COVID-19, and that recovered asymptomatic carriers had either negative or very low anti-S titers compared to those who recovered from severe COVID-19 [19]. This was supported by the results of Shirin et al. [20] where lower anti-S levels were reported following SARS-CoV-2 infection in asymptomatic patients compared to mildly symptomatic ones.

The mechanism underlying the association between anti-S titers and the severity of SARS-CoV-2 infection remains unclear. However, two theories were proposed; either excessive inflammation which leads to the overproduction of antibodies, or high viral loads with a high amount of viral antigens which contribute to a stronger serological response with higher anti-S titers [19].

The direct correlation between young age and asymptomatic state in COVID-19 participants in this study was consistent with the results of other studies. Al-Rifai et al. reported that younger COVID-19 cases were more likely to be asymptomatic and healthier than older cases [14]. A similar association between age and an asymptomatic state was also demonstrated in a study conducted in Wuhan, China [21]. This may be attributed to the absence of comorbidities and a healthy immune system in younger age groups.

In our work, the positive correlation between anti-S titers and the number of symptoms (Fig. 1) was paralleled with a direct correlation of both with the duration needed for clinical improvement (Table 4). This might explain the significant short duration needed for improvement in younger age participants as seen in this study. Similarly, other researchers reported that the severity of initial symptoms is a significant predictor of disease duration [22]. Moreover, in our study, the number of symptoms was found to affect COVID-19 diagnosis, where having more than 3 symptoms was significantly associated with “being diagnosed”, by any means (clinically, radiologically, or by laboratory tests), with a sensitivity of 70.2% [95% CI 61.9–77.6%], and specificity of 73.7% [95% CI 70.2–77.0%]. This indicates that patients with fewer symptoms are more likely to be undiagnosed, yet, still they are a source of infection to others. Health education for the public is required regarding the role of asymptomatic individuals as well as oligosymptomatic ones in spreading the infection to others, to interrupt the infectious cycle.

In this work, there was an inverse association between, on the one hand, less duration of contact with the public (indicated by longer periods of online working/studying) and on the other hand, COVID-19 symptoms. This denotes that contact with the public increases the risk of exposure to COVID-19 patients and carriers which might account for increasing viral load and subsequent immune response (represented by the anti-S titer). Hence, participants working online were less prone to being symptomatic. This was also reported in other studies [14, 23], and it emphasizes the role of social distancing, lockdown, and travel restrictions imposed by governments to combat the pandemic.

A direct correlation between higher BMI among adults, COVID-19 symptoms, and anti-S titer was demonstrated in our study (Table 4). Similarly, obesity was associated with severe SARS-CoV-2 infection and higher neutralizing COVID-19 antibody titer in the data published by the Mount Sinai Health System in New York City [24]. Similar results were reported in another cohort study conducted in Thailand on 147 adult patients with confirmed COVID-19, where obesity was associated with severe pneumonia and adverse outcomes of SARS-CoV-2 infection [25]. The underlying mechanisms connecting obesity and severe COVID-19 could be immune dysregulation, comorbidities, and an impaired respiratory system in obese patients [26]. Increased antibody titer following SARS-CoV-2 infection in patients with higher BMI may indicate an exaggerated immune system and a higher tendency towards severe symptoms owing to abnormal production of cytokines from the adipose tissue. This, in turn, creates an imbalance in pro-inflammatory and anti-inflammatory cytokines in those patients [27].

People living in slums are among the most vulnerable communities to SARS-CoV-2 infection. Unfeasibility of infection control measures such as hand washing facilities, social distancing, and wearing masks as well as poor access to health services are more common within these settings. In this study, participants from slum areas had approximately twofold greater risk than those from urban areas for being symptomatic COVID-19 (aOR = 1.845, *p* = 0.037). In contrast, the majority of COVID- 19 participants enrolled in a study conducted in a large slum in South India were asymptomatic [28].

Participants working as healthcare workers and those working in client-facing occupations had nearly two and one folds greater risk of being symptomatic than those not working or not facing clients in their jobs (aOR = 2.111, *p* = 0.014 and aOR = 1.231, *p* = 0.026), respectively. In addition, a significantly higher proportion of symptomatic participants were among those working or studying for only 1–3 days/week online compared to those working or studying 6–7 days/week online. The protective effect of online working/studying was confirmed by the multivariate analysis where the least number of days for working or studying online (1–3 days/week) was associated with nearly one and a half greater risk for symptomatic SARS-CoV-2 infection compared to full time online working or studying. During the early waves of the pandemic, online education was adopted by the Egyptian government as a measure to slow down the spread of infection.

Regarding wearing masks/social distancing or using soap for hand washing, the highest prevalence of symptomatic COVID-19 was among those who never wore masks or practiced social distancing, or used soap for hand washing. They had approximately 1.5 to 3 times increased risk of being symptomatic (aOR = 1.566, *p* = 0.019 and aOR = 2.851, *p* = 0.018, respectively).

Considering the effect of comorbidities on acquiring symptomatic SARS-CoV-2 infection, a significantly higher proportion of participants suffering from lung, kidney, or autoimmune diseases were symptomatic. While both kidney and autoimmune diseases did not have an increased risk in multivariate analysis than those without, participants suffering from lung diseases (COPD/ asthma) were found to be more than 2 folds- at risk than those without such diseases (aOR = 2.270, *p* = 0.006). Similarly, in a meta-analysis study on the predictive symptoms and comorbidities for severe COVID-19, Jain and Yuan reported that COPD patients were particularly vulnerable, and those with cardiovascular diseases and hypertension were also at a high risk of severe illness. In their study, COPD was the most strongly predictive comorbidity for both severe disease (pOR 6.42, 95% CI 2.44–16.9) and ICU admission (pOR 17.8, 95% CI 6.56–48.2) [29].

In our study, participants who had blood group “A” had almost a 2-times higher risk of being symptomatic compared to those with group “O” (aOR = 1.851, *p* = 0.015). This result was consistent with that of a genome-wide association study which identified a relationship between polymorphisms in the genes encoding the ABO blood group and respiratory failure from COVID-19 [30]. The association of the type A blood group with a high risk of acquiring symptomatic and severe disease compared to the low risk associated with the type O blood group was also demonstrated in other studies [30, 31].

"Exercising" was a significant protective factor against being symptomatic of COVID-19. Participants who never exercised had a 1.5 increased risk of acquiring symptomatic COVID-12 compared to those who exercised consistently (aOR = 1.597, *p* = 0.039). Similarly, a study revealed that cases with COVID-19 who were consistently inactive had a greater risk of hospitalization, admission to the ICU, and death due to COVID-19 compared to individuals who were consistently doing physical activity. Consistently meeting physical activity guidelines was strongly associated with a reduced risk for severe COVID-19 outcomes among infected adults [32]. This may be a result of cardiovascular fitness and decreased obesity in those consistently active. Thus, physical inactivity can be considered one of the modifiable risk factors for COVID-19.

## Conclusions

The prevalence of asymptomatic COVID-19 cases among the studied participants was high but still within the reported range (34.9%). Living in slums, working in facing rooms rather than online lack of infection control measures as well as deficiency of regular exercise were predictors for symptomatic state among SARS-CoV-2 infected individuals. Such modifiable risk factors should be addressed to reduce the severity of symptoms among COVID-19 cases.

## Data Availability

For confidentiality reasons, raw data are not publicly available. They are stored in encrypted excel sheet form with accessibility to only the corresponding author. They might be available upon reasonable request.
